# The societal benefits of reducing six behavioural risk factors: an economic modelling study from Australia

**DOI:** 10.1186/1471-2458-11-483

**Published:** 2011-06-21

**Authors:** Dominique A Cadilhac, Anne Magnus, Lauren Sheppard, Toby B Cumming, Dora C Pearce, Rob Carter

**Affiliations:** 1 Deakin Health Economics, Deakin University, Burwood 3125, Victoria, Australia; 2 National Stroke Research Institute, Heidelberg 3084, Victoria, Australia; 3 The University of Melbourne 3010, Parkville, Victoria, Australia

## Abstract

**Background:**

A large proportion of disease burden is attributed to behavioural risk factors. However, funding for public health programs in Australia remains limited. Government and non-government organisations are interested in the productivity effects on society from reducing chronic diseases. We aimed to estimate the potential health status and economic benefits to society following a feasible reduction in the prevalence of six behavioural risk factors: tobacco smoking; inadequate fruit and vegetable consumption; high risk alcohol consumption; high body mass index; physical inactivity; and intimate partner violence.

**Methods:**

Simulation models were developed for the 2008 Australian population. A realistic reduction in current risk factor prevalence using best available evidence with expert consensus was determined. Avoidable disease, deaths, Disability Adjusted Life Years (DALYs) and health sector costs were estimated. Productivity gains included workforce (friction cost method), household production and leisure time. Multivariable uncertainty analyses and correction for the joint effects of risk factors on health status were undertaken. Consistent methods and data sources were used.

**Results:**

Over the lifetime of the 2008 Australian adult population, total opportunity cost savings of AUD2,334 million (95% Uncertainty Interval AUD1,395 to AUD3,347; 64% in the health sector) were found if feasible reductions in the risk factors were achieved. There would be 95,000 fewer DALYs (a reduction of about 3.6% in total DALYs for Australia); 161,000 less new cases of disease; 6,000 fewer deaths; a reduction of 5 million days in workforce absenteeism; and 529,000 increased days of leisure time.

**Conclusions:**

Reductions in common behavioural risk factors may provide substantial benefits to society. For example, the total potential annual cost savings in the health sector represent approximately 2% of total annual health expenditure in Australia. Our findings contribute important new knowledge about productivity effects, including the potential for increased household and leisure activities, associated with chronic disease prevention. The selection of targets for risk factor prevalence reduction is an important policy decision and a useful approach for future analyses. Similar approaches could be applied in other countries if the data are available.

## Background

About one third of the total burden of disease and injury in Australia can be attributed to 14 behavioural, physiological and environmental/social risk factors [1]. Begg and colleagues have estimated that tobacco is responsible for the greatest disease burden in Australia (7.8% of total burden), followed by high blood pressure (7.6%), high body mass (7.5%), physical inactivity (6.6%), and high blood cholesterol (6.2%). Therefore, reducing disease risk factors, even by small amounts, would have a major impact on improving health and productivity in Australia. Recent international research has highlighted the potential to avert a large number of deaths due to chronic disease by delivering relatively low cost population-based interventions targeting salt intake and tobacco use [2] and offering combination pharmacotherapy for cardiovascular disease [3]. However, funding for health promotion initiatives remains limited. For example, in Australia about 2.5% of the total health budget is used to support public health programs [4]. The majority of these funds are allocated to screening, risk assessment and immunisation programs.

Quantifying the potential benefits of disease prevention is, therefore, an important exercise in positioning for additional funding for reducing chronic diseases. Moreover, government and non-government organisations are showing a growing interest in the productivity effects from reducing chronic diseases. This is because government will benefit through future savings in health care expenditure on treatments for disease and from increased taxation transfers via larger individual incomes. Businesses will benefit from reduced absenteeism from work and less recruitment and training costs associated with replacing staff who die or retire prematurely due to ill health. Individuals benefit from increases in income, reduced absenteeism from work and/or time spent out of their roles at home and improved quality of life from reduced levels of ill health.

We aimed to estimate the 'health status' and 'economic' benefits of reducing the current prevalence of six specified behavioural risk factors to a ***feasible ***target level for: ***tobacco smoking; inadequate fruit and vegetable consumption; high risk alcohol consumption; high body mass index; physical inactivity; and intimate partner violence***. These six risk factors were selected by a nationally representative Advisory Committee on the basis that they signify priority public health issues. The Advisory Committee comprised a broad range of experts from government and non-government sectors including health economics, health promotion and policy. The reported current prevalence of these nominated risk factors varies depending on how they are measured. Over the last 20 years there has been an average decline in tobacco smoking of about 1% per annum with estimates for current daily smokers at about 23% of the population [5]. The number of people consuming fruit and vegetables in Australia at a level required to avoid ill health is inadequate. Data from the 2004-05 National Health Survey (NHS) indicated that 46% of Australian adults eat one serve or less of fruit per day and that 40% of Australian adults eat two serves or less of vegetables per day [5]. With regards to alcohol consumption, about 13% of Australian adults drink at 'risky' or 'high risk' levels long term (> 4 standard drinks for men and > 2 standard drinks for women per day). Overweight and obesity estimates vary mainly because people under-report their weight. NHS data provides evidence that 43% of men were overweight and 19% obese, while in women, 28% were overweight and 17% were obese [5]. Direct measurement data from a comparable general population sample suggest that 27% of Australian adults are obese [6]. Overall, 70% of adults were sedentary or had a low exercise level [5]. The data for intimate partner violence are also subject to under-reporting and measurement differences, with estimates of women in Australia who have experienced violence by a current and/or former partner ranging from 10% to 27% [7,8].

## Methods

**Figure 1:**
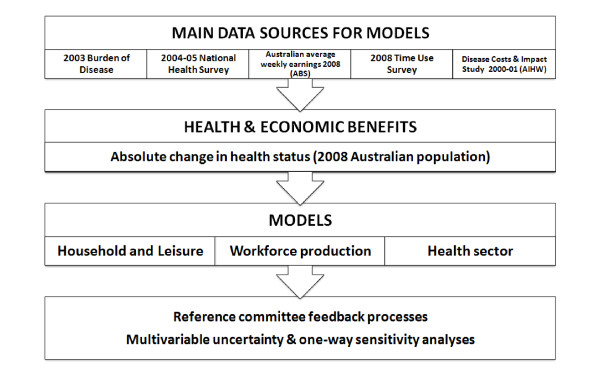
**Schematic diagram of models and data inputs**. ABS: Australian Bureau of Statistics. AIHW: Australian Institute of Health and Welfare. UQ: University of Queensland.

The following provides a summary of the main methods and assumptions applied in this study. Since the methods for this research are beyond what can be described in a single paper, a full technical report can be accessed at http://www.vichealth.vic.gov.au/Publications/Research/Health-and-economic-benefits-of-reducing-disease-risk-factors.aspx. A schematic diagram of how the economic modelling was undertaken and the relationship between sub models is provided in Figure 1.

Current risk factor prevalence estimates were largely based on data from the 2003 Australian Burden of Disease (BoD) study [8]. We used standard definitions for the six risk factors and the comparator non-exposed status to analyse the NHS data on workforce participation (Table 1). The comparator *'non-exposed' *status was based on people with the risk factor achieving a *'prior exposure' *status rather than *'never' *exposed status (e.g. a smoker was compared to an ex-smoker, not a never smoker).

**Table 1:** Definitions of risk factors*

Alcohol consumption	Long term high risk alcohol consumption: Greater than 75 mls of alcohol consumed per day for men, and greater than 50 mls of alcohol consumed per day for women.
	Long term low risk alcohol consumption: Less than 50 mls of alcohol consumed per day for men, and less than 25 mls of alcohol consumed per day for women.
High body mass index	Obese or overweight: BMI greater than 25, based on self-reported height and weight.
	Normal weight: BMI less than 25, based on self-reported height and weight (including underweight).
Inadequate fruit and vegetable consumption	Inadequate fruit and vegetable consumption: Consumption below the recommended minimum of 2 serves fruit and 5 serves vegetables daily.
	Adequate consumption: Consumption at or above the recommended minimum of 2 serves fruit and 5 serves vegetables daily.
Intimate partner violence	High psychological distress has been used as a proxy for current exposure to intimate partner violence: High or very high levels of psychological distress (score 22-50 on the Kessler Psychological Distress Scale -10).
	Moderate psychological distress has been used as a proxy for past exposure to intimate partner violence: Moderate levels of psychological distress (score 10-21 on the Kessler Psychological Distress Scale -10).
Physical inactivity	Inactive: Sedentary or low activity level.
	Active: Moderate to high activity level (i.e. 3 sessions of at least 20 to 40 minutes vigorous exercise or 5 sessions of at least 30 minutes moderate exercise per week).
Tobacco smoking	Current smokers: Persons who smoke tobacco on a regular or irregular daily basis.
	Ex-smokers: Persons who no longer smoke on a regular or irregular basis.

*These definitions were used to categorise cases in the National Health Survey to assess differences in workforce behaviour. The impact on health outcomes associated with these risk factors in our models were based on mortality and morbidity rates by age group and gender in the determination of age/sex specific population attributable fractions taken from the 2003 Australian Burden of Disease Study [1]. BMI: Body mass index.

The risk factor prevalence scenarios were modelled separately for each risk factor using best available evidence following an extensive literature review to inform decisions on what constituted realistic and feasible reductions. The methods and results for the smoking risk factor have been published recently [9]. The decision on what constituted 'best available' evidence for the realistic reductions varied between the risk factors. In the estimations for alcohol, tobacco and intimate partner violence it was agreed that the feasible reductions should be modelled against attainment of prevalence levels observed in a comparable country (referred to as an 'Arcadian' ideal). Comparing equivalent countries to identify an *Arcadian ideal *has been used in other studies [10-12]. In contrast, for inadequate consumption of fruit and vegetables, high BMI and physical inactivity, a consensus approach informed by best available evidence was preferred (Table 2). For these risk factors, the Advisory Committee agreed that international comparisons were too problematic, mainly due to country specific socio-economic and cultural variations.

**Table 2:** Summary of selected feasible reductions for each risk factor

Risk factor	Method	Attributable			Change		Reference/Source
			
		Australia	Comparator		Feasible	Progressive*	
Intimate partner violence (prevalence %)	Arcadian ideal	27	Denmark: 22		↓5	↓2.5	[7]

High risk alcohol consumption (litres/capita/year)	Arcadian ideal	9.8	Norway: 6.4		↓3.4	↓1.7	[8]

Tobacco smoking (prevalence %)	Arcadian ideal	23	California: 15		↓8	↓4	[9]

Physical inactivity (prevalence %)	Evidence based consensus	70	60		↓10	↓5	[10]

Inadequate fruit and vegetable consumption (grams/day/person)	Evidence based consensus	503	675		↑172	↑86	External consultation and Australian guidelines.

High body mass index (prevalence %)	Evidence based consensus	27	24		↓3	↓1.5	Dutch intervention study; external consultation; National Preventative Health Taskforce

*Progressive is half-way to achieving the feasible target

The approaches we used for target setting ensured realistic estimates (Table 2) given the evidence for each risk factor that would be relevant to policy-makers. Irrespective of which approach was used as the basis of the 'feasible' reduction in risk factor targets, systematic methods and data sources were used for the analysis.

Two different absolute levels of achievement of reductions in risk factor prevalence were modelled for each risk factor using threshold analysis principles. The ***feasible ***target (i.e. what we ***might achieve ***based on current knowledge in the medium/long term) and a ***progressive 'half-way' target ***(reflecting attainment of half of the reduction in risk factor prevalences desired) were agreed upon and then modelled (Table 2). A schematic diagram of our modelling approach is presented in Figure 1. We did not include time lags associated with risk reversibility and the consequent reduced incidence of diseases. Rather, we assumed an immediate impact in the cohort of interest based on the characteristics of the reference cohort and detailed epidemiological data available by age and gender from the 2003 Australian BoD study [8]. This appears reasonable given the growing evidence of rapid benefits following population-wide policy interventions [13].

To emphasise the magnitude of our findings in relation to the status quo if no changes in the prevalence of these risk factors occur, we report the current attributable level of disease burden associated with these risk factors. This is equivalent to the amount of ill health that could be averted if the risk factors could be fully eliminated from the Australian population. We then provide the potential benefits if the reduction targets are achieved. The 'health status' benefits were measured as changes in the associated incidence of diseases (for example stroke, cancers and heart disease), as deaths and as Disability Adjusted Life Years (DALYs) associated with the risk factor reduction. The 'economic' benefits were measured as changes in workforce participation rates, absenteeism and early retirement from the workforce, as well as days of increased household and leisure activities that could be associated with improvements in health status. These economic benefits were then valued in 2008 Australian dollars as potential opportunity cost savings. The opportunity cost savings from preventable disease should not be considered as immediately realisable cash savings.

### Data sources

Data for the risk factors of interest (e.g. prevalence estimates by age and gender, population attributable risk fractions [PAFs], etc), health status estimates (incident cases of disease, deaths) and full DALYs were obtained using the 2003 Australian BoD data files [8] provided for use in this study. The 2000-01 Disease Costs and Impact Study (DCIS) [14] Excel files, which have been created using BoD classification nomenclature, were used to estimate the change in health sector costs associated with our risk factors. DCIS is a descriptive study outlining the health sector costs of diseases using a systematic costing methodology. However, DCIS does not provide cost information for individual risk factors. To estimate the health sector costs for the risk factors, we back calculated the attributable portion of total health sector costs associated with the risk factors. Although not a perfect approach, this method offered greater consistency, reliability and internal validity for this study. The 2001 costs were inflated by the total health price inflation factor of 1.239 to approximate these costs in 2008 reference year dollars [15].

Demographic data and information on the nominated health risk factors, employment status, and health-related actions were obtained from the 2004-2005 NHS Confidentialised Unit Record Files (CURF), with the approval of the Australian Statistician, Australian Bureau of Statistics (ABS). The NHS has self-reported information about the health status of Australians, use of health services, and other health-related aspects of lifestyle. Characteristics of the Australian population were estimated from the CURF data, with weights (expansion factors) assigned to individual responder's records consistent with the sampling strategy [5]. All data sources were de-identified and ethics approval was not required for this study.

Direct risk factor information (e.g. current smokers and ex-smokers) from the NHS to estimate differences in workforce productivity and days out of role due to ill health for household productivity and leisure time was available for all risk factors except intimate partner violence. For this study, we used the workforce behaviours of persons with high psychological distress as an approximation of the behaviours of women exposed to intimate partner violence. This was justified because anxiety and depression are the most common health-related outcomes of intimate partner violence [8]. The analysis of intimate partner violence was only attempted for women given the paucity of information in men [8].

To calculate household production and leisure time, we used data files from the 2006 Time Use Survey [16] downloaded from the ABS website. Current average wages were obtained from the ABS and/or published government pay scale summaries [17,18].

### Data analyses

**Figure 2:**
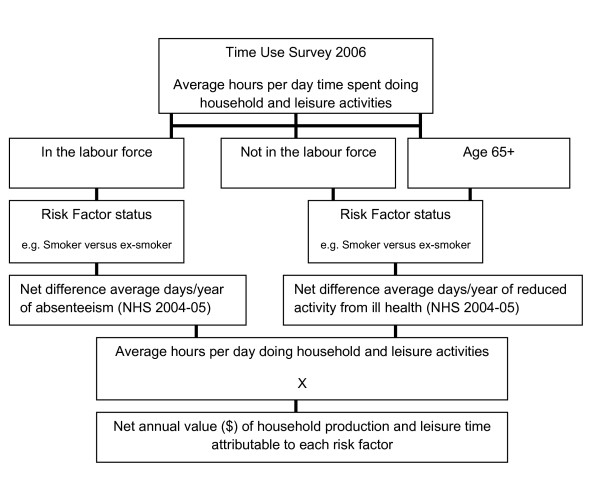
**Overview of household contribution, leisure time and household production calculation, calculated for each gender and workforce combination**. NHS: National Health Survey.

We developed population simulation models in Excel (Microsoft Office, 2003) and applied threshold analysis principles to determine the potential lifetime benefits of reducing the prevalence of behavioural risk factors for the 2008 Australian adult cohort (aged 15 years and over; about 17 million people). The simulation models for people in the workforce were developed from previous work undertaken by Deakin Health Economics for the Victorian Department of Treasury and Finance [19]. Additional models were developed for this study to estimate the lost leisure and household production associated with diseases attributable to the risk factors of interest (Figure 2).

We applied methods to correct for the joint effects of multiple risk factors, because most people in the Australian population have more than one of the risk factors of interest regardless of workforce status. That is, where two or more risk factors contributed to a disease outcome a statistical adjustment was required to avoid overestimating benefits. The joint effects correction was based on methods outlined in the 2003 Australian BoD study [8] and by the World Health Organization [20]. In brief, a formula known as the ***joint population attributable risk fraction ***(joint PAF) is used. This formula is based on the assumption that health risks are biologically independent (although it is acknowledged that this is not always the case) [20]. This assumption allows the joint PAF for *n *number of risks to be expressed as joint PAF = 1-(1-PAF_a_)*(1-PAF_b_)*(1-PAF_c_), etc [8].

Where cost data were taken from an alternative year, these were adjusted to 2008 by applying published total health price inflation indices [15]. The time horizon for economic benefits was based on a lifetime perspective for the 2008 Australian population cohort. Benefits determined for the rest-of-life for the 2008 cohort were based on current Australian life expectancy estimates for men and women [21]. The remaining average years of life were determined by subtracting the average age of each reference group (working, not in the labour force and over 65 years of age) according to gender. A 3% discount rate for lifetime benefits was used to match the rate chosen in the Australian BoD studies. It is also the rate of discounting recommended by a consensus panel of health economists in the US [22] and is commonly used in Australian health economics studies [23,24].

### Workforce Production Gains model

In this study, workforce productivity effects were estimated to reflect changes in workforce participation and absenteeism associated with the health status of people aged 15 to 65 years (i.e. work ages until retirement) and determining the net health benefits for the working years of life. Workforce participation was defined as people working part-time, full-time or looking for work. 'Presenteeism', which is a measure and valuation of persons at work who are less productive because of ill health, was not included.

There are two main techniques which have been most frequently used in economic evaluation to measure and value productivity gains and losses: the Human Capital Approach (HCA) and the Friction Cost Approach (FCA). In brief, HCA counts all future income lost from an individual who leaves the workforce due to death or disability, whereas the FCA assumes individuals will be replaced after a specified period and thus productivity losses to society will be reduced. However, there is still debate in the literature about which method is preferable; see Koopmanschap and Rutten [25] and Liljas [26] for alternative views. The HCA remains the dominant methodology utilised to measure productivity costs in many of the published cost of illness studies. However, we believed the FCA method was more appropriate for our research question, that is, *for estimating production gains/losses in the general economy*. For this question it was important for us to take into account the fact that businesses will adjust to short term and long term absences. In this present study, the friction period was assumed in the Workforce Production Gains model to be 3 months [25,27] and varied to 6 months in sensitivity analysis. In contrast, the HCA is more suited to answering a different research question, that is, placing a monetary value on human life, where the total forgone income stream due to premature death provides a sensible ceiling estimate. Given the acknowledged differences of opinion regarding which method may be more appropriate, as a sensitivity analysis we also calculated workforce production effects using the HCA.

### Household Production and Leisure Time model

In this study it was considered important to capture aspects of productivity that go beyond those participating in the paid workforce. Household production was defined as the hours of time spent performing non-paid household duties such as cooking, shopping, cleaning, child care and maintenance. These duties were valued by assuming the 'replacement cost method' (i.e. the services would be purchased commercially when a person in the household was unable to perform them through ill health). The unit prices for household production were based on the average wage rates for commercially available domestic services and child care (Additional file 1, Table S1). Leisure time comprised social and community interaction and recreation and leisure activities. The value of increased leisure time was determined using an 'opportunity cost' method by applying one third of the average weekly earnings [28] for men and women reported by the ABS for 2008 (Additional file 1, Table S1). It is acknowledged that the value of leisure time may differ for a number of reasons, such as when leisure time is scarce an individual may value it more. Therefore, we varied the unit prices from 25% up to 50% of the average weekly earnings in uncertainty analyses.

An overview of how household production and leisure time was calculated is provided in Figure 2. Estimates of leisure and household production hours per day from the ABS 2006 Time Use Survey were applied to the surveyed days of absenteeism or reported days out of role associated with illness by each risk factor categorised according to gender and workforce status (working, not in the labour force, retired) taken from the 2004-05 NHS. The net difference in days of household and leisure time between persons with and without the risk factors was then valued using relevant unit prices (see Additional file 1, Table S1).

### Health Sector cost estimation

The DCIS data provided information for one year only and included all health sector costs associated with the treatment of all incident and prevalent cases of disease based on episodes of care [14]. We did not attempt to model lifetime health expenditure costs for disease events from DCIS data because the data were cross-sectional. Comprehensive, longitudinal health sector costs data from cohort studies were unavailable in Australia. Therefore, we assumed that the annual cost of treating risk factor-related diseases was a representative approximation that could be used to model lifetime costs of treating incident cases.

In some instances it was more difficult to estimate the health sector costs associated with risk factors. This included several diseases related specifically to alcohol use (e.g. pancreatitis, gall bladder and bile duct disease, inflammatory heart disease and alcohol dependence and harmful use); tobacco use (e.g. chronic obstructive pulmonary disease, lower respiratory tract infections, age related vision disorders, etc) and intimate partner violence (such as, sexually transmitted diseases). Where we did not have access to the DCIS files for these diseases associated with the risk factors, we used an indirect method by estimating the proportion of total DALYS for these diseases attributed to each risk factor and then multiplying this proportion by the total costs for the published health expenditure category that these diseases belong to e.g. mental disorders, respiratory, digestive system, etc [14]. This was an important benefit of having the detailed 2003 Australian BoD files for this work and the ability to confidently use the DCIS results since the same disease classification is used.

### Uncertainty and sensitivity analyses

Multivariable probabilistic uncertainty analyses were undertaken using @RISK software version 4.5 for Excel [29]. A minimum of 4,000 simulations using Monte Carlo sampling were used to estimate a mean, median and 95% uncertainty interval around each outcome parameter generated. Input variables were modelled as known distributions rather than single values where quantifiable uncertainty existed (e.g. surveyed parameters and life-years remaining) (see Additional file 1, Table S2). One-way sensitivity analyses were also undertaken whereby single input parameters were varied, such as the use of the HCA.

## Results

In total, it is estimated that about AUD9,000 million could have been saved if each of the six risk factors had been eliminated from the Australian population in 2008 (Table 3). In other words, if the current attributable level of disease burden associated with these risk factors could be eliminated from the Australian population, there could be AUD3,540 million in workforce, household and leisure production costs (95% Uncertainty Interval [UI]-AUD213, AUD7,444) and AUD5,329 million in health sector costs avoided (UI not estimated for these latter costs due to reliable data being unavailable). This is the upper limit of potential opportunity cost savings that could be achieved assuming risk was fully reversible and if funding was available to fully implement effective interventions. The impact of the joint effects correction was to approximately halve all benefits (e.g. DALYs, deaths avoided, etc), with the exception of health sector costs.

**Table 3:** Total potential attributable opportunity cost savings

	Uncorrected individual risk factors						Combined risk factors
**Attributable**	**IPV** **($mill)**	**High risk alcohol** **($mill)**	**Poor diet** **($mill)**	**Physical inactivity** **($mill)**	**Tobacco smoking** **($mill)**	**High BMI** **($mill)**	**Corrected for JE** **($mill)**

Total production (FCA)	1,801	1,224	63	1,135	1,215	742	3,540
Health sector offsets	207	2,275	206	672	1,412	812	5,329
*Total*	2,008	3,498	269	1,807	2,627	1,554	8,869

The data in this table represents the current estimated production and health sector costs based on current risk factor prevalence estimates. FCA: Friction cost approach for valuing workforce productivity losses; IPV: Intimate Partner Violence; BMI: Body mass index; JE: adjusted for joint effects of risk factors; $mill: Australian dollars in millions.

**Figure 3:**
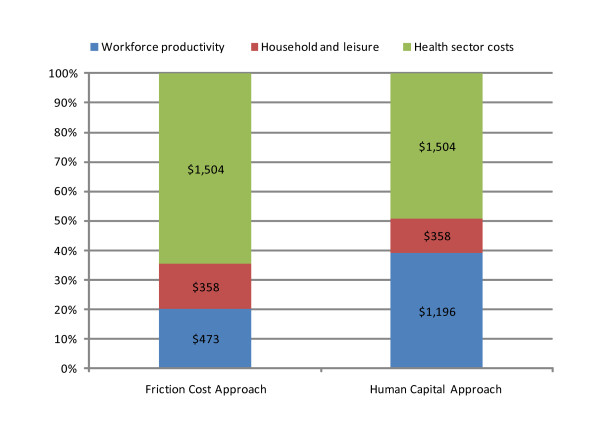
**Differences in the relative contribution of workforce production effects estimated using the Friction Cost Approach versus the Human Capital Approach**. Dollar values are Australian dollars in millions.

If *feasible *reductions in the risk factors were achieved concurrently, we estimated that total potential opportunity cost savings of AUD2,334 million (workforce, household and leisure production costs of AUD830 [95% UI -AUD109, AUD1,843] and AUD1,504 million in health sector costs) over the lifetime of the 2008 Australian adult population were achievable (Table 4). Based on our 2008 population estimate of 20,937,986 people [30] this would be equivalent to $112 per capita in opportunity cost savings. These results were obtained from an increase in 5 million days in workforce production from reduced absenteeism; 626,000 more days for household production activities; and 529,000 increased days of leisure time, associated with 161,000 new cases of disease being prevented and 6,000 fewer preventable premature deaths. Moreover, we estimated that there would be 95,000 fewer DALYs (this is equivalent to 23% of the current attributable DALY burden associated with these risk factors or about 3.6% of total DALYs estimated for Australia). Use of the HCA doubled the relative contribution of workforce production as a proportion of the total production gains (Figure 3). The largest potential savings could be gained from reductions in alcohol consumption followed by reductions in tobacco smoking, intimate partner violence, physical inactivity, BMI and lastly from increases in the consumption of fruit and vegetables.

**Table 4:** Financial outcomes of all risk factors if feasible targets achieved, corrected for joint effects

All 6 risk factors		95% Uncertainty Interval	
AUD millions	Mean	Lower Limit	Upper Limit
***Potential opportunity costs FCA ***			
Production gains/(losses)	473	(2)	1,155
Recruitment/training costs	79	n/a	n/a
Leisure based production	110	(361)	602
Home based production	248	(69)	568
***Total production FCA***	***830***	**(*109*)**	***1,843***
Health sector offsets	1,504	1,504	1,504
**Total Opportunity Cost Savings FCA**	**2,334**	**1,395**	**3,347**
			
**Sensitivity analysis using Human Capital Approach (HCA)**			
***Financial Outcomes HCA***			
Production gains/(losses)	1,196	(648)	3,070
Leisure based production	110	(361)	602
Home based production	248	(69)	568
***Total production HCA***	***1,553***	**(*435*)**	***3,569***
Health sector offsets	1,504	1,504	1,504
**Total Opportunity Cost Savings HCA**	**3,057**	**1,069**	**5,073**

No probabilistic uncertainty analysis was conducted for health sector offsets because we were unable to quantify uncertainty for these point estimates. Taxation is treated as a transfer payment and should not be added to production effects or health sector offsets. HCA: Human Capital Approach; FCA Friction Cost Approach (preferred conservative estimate). Leisure and home based production estimates are based on persons 15+ years. Production gains/(losses) and taxation effects are based on persons 15-64 years. Recruitment and training costs are included in production gains/losses using the FCA, but not counted using the HCA. Values are net present value using a 3% discount rate. Numbers in brackets ( ) indicate the possibility of losses resulting from achieving the target, rather than gains.

If broadly half the benefits were achieved (progressive target), total opportunity cost savings of AUD1,171 million (workforce, household and leisure production costs of AUD419 95% [UI -AUD55, AUD923] and AUD752 million in health sector costs) over the lifetime of the 2008 Australian adult population were achievable. There would be 48,000 fewer DALYs; 80,000 less new cases of disease; 3,000 fewer deaths; an increase in 2.4 million days in workforce participation; and 264,000 increased days of leisure time.

Further details are provided in the full technical report (http://www.vichealth.vic.gov.au/Publications/Research/Health-and-economic-benefits-of-reducing-disease-risk-factors.aspx).

## Discussion

This is a novel study where there has been a systematic assessment of the benefits of concurrently reducing six important behavioural risk factors in the Australian community. These six behavioural risk factors lead to avoidable illnesses such as stroke, diabetes and cancer for millions of Australians. The potential benefits were modelled as lifetime estimates for a single (2008) population cohort. If the ideal targets for reductions in risk factor prevalence could be achieved then substantial opportunity costs savings were found (AUD2,334 million NPV at 3% discount rate using the FCA method with correction for joint risk factor effects). These summary estimates are particularly useful where preventative public health initiatives compete against acute disease treatment interventions for scarce health sector resources. This is because the opportunity costs of preventing disease have a much larger impact on a population compared to intervention options for treating cases of diseases alone. Moreover, these estimates provide a fuller picture of what might be achieved in terms of production gains because of the application to all members of society and not just people in the workforce. Capturing important household and leisure activities, such as caring for families, preparing meals and cleaning are increasingly recognized as essential to maintaining a work-life balance and being healthy, yet are rarely accorded their economic worth.

We used conservative estimates to value total productivity effects based on common approaches in economics (e.g. the use of average 2008 wage rates for household based on replacement costs for commercial services, but for workforce production we used age and gender specific average 2008 wage rates). In the financial year 2006-07, Australia's health expenditure totalled AUD94.0 billion, representing 9.0% of gross domestic product (GDP) [15]. Therefore, our health sector cost savings, which represent an annual estimate, represents approximately 2% of this total annual health expenditure. Although the costing method used to estimate health sector costs may cause over- or under-estimation of total costs, the costing methodology for each disease group is undertaken using a systematic approach and by the same BoD nomenclature that we have used to determine the association between risk factors and diseases to facilitate comparisons between the risk factors.

It is difficult to compare our findings with previous Australian and international literature for three main reasons. First, most research has focussed on quantifying economic costs and benefits for a single risk factor [11,31-34]. Where multiple risk factors have been considered, the analysis has been limited to workforce production [35] and without correction for joint effects. Second, significant methodological differences between the current study and previous literature limit a direct comparison of findings. Previous researchers have variously chosen to model incident or prevalent cases; included different definitions of risk factors and disease, unit costs and inclusion/exclusion criteria; and may have focussed on disease-specific health sector costs of a risk factor. Third, household production and leisure time has generally been excluded from previous analyses. Therefore, our findings provide important new information, especially for policy decision-making regarding the value for investment in health promotion initiatives from a societal perspective.

The main strength of the analyses presented is the use of the best available evidence to provide a comprehensive and consistent examination of the benefits that may be possible where policy initiatives concurrently address feasible reductions in the prevalence of multiple risk factors. Another strength is the important correction downward to the estimates for the joint effects of multiple risk factors that contribute to the same diseases. Our joint effects correction prevents overestimation of total potential benefits. The use of uncertainty analyses to address the limitations of our data provides confidence in the main study finding, indicating that total potential opportunity cost savings are 95% likely to fall between AUD1,395 million and AUD3,347 million.

The main limitation of this project is the reliance on cross sectional, self-reported data from the NHS and the Time Use Survey to identify the association between the risk factors and reduced productivity due to ill health. Assuming causality in the absence of robust longitudinal data means that our results must be regarded as broadly indicative. This is because self reported data are less reliable than actual measurement data, since persons exaggerate or understate, fail to remember accurately, misunderstand questions and diseases, or simply misreport information. However, the evidence of a causal relationship between the risk factors investigated and the associated disease outcomes is sound and our assumption that a risk factor will lead to ill health and losses in productivity, despite some anomalies from using cross-sectional data, was valid.

Overall, the direction of bias from reliance on cross sectional, self-reported data is likely to underestimate our results since, in some cases, people without a risk factor were less productive than those with a risk factor which is reflected in the 95% uncertainty interval of the estimates. For example, men with high risk alcohol consumption and male current smokers were more active than their counterparts (i.e. low risk alcohol consumers or ex-smokers). The implication is that ex-smokers have more reduced days of activity as do low risk alcohol consumers who are not working. This led to some of the estimated benefits being negative when the prevalence of the risk factor was reduced (data not shown). This may be a plausible assertion and could, in part, be due to some people not in the workforce or over 65 years of age already being unwell and not working because of health effects, which may have also resulted in them becoming an ex-smoker or reducing their alcohol consumption. For example, people who consumed alcohol at levels associated with high risk may avoid leisure time activities where they would be encouraged to consume alcohol. Unfortunately, more detail about these cases is unavailable because the best available Australian data for this project were cross-sectional. This highlights an area for more research. Nevertheless, when the estimates are summed for men and women for both household production and leisure time, overall, there was a large attributable cost associated with smoking and high risk alcohol consumption, which could be reversible.

Another limitation is the paucity of evidence on the effectiveness of specific interventions to inform judgements about feasible reductions in risk factor prevalence. Furthermore, the selection of feasible targets was contentious, in particular for high BMI and inadequate fruit and vegetable consumption where targets may appear optimistic. This highlights future research requirements in this area to support further decision analytic modelling for health promotion. Lastly, since people are not questioned in the NHS on the issue of intimate partner violence, we relied on surveyed levels of psychological distress amongst women as a surrogate measure [5]. The BoD studies have identified that depression and anxiety are the largest components of illness attributable to intimate partner violence [8]. The Kessler 10 score used in the NHS was considered to have adequately captured depression and anxiety in the general population for this study [36]. The impact of this approach on our findings is a probable underestimate of the health and economic costs of intimate partner violence.

We have provided conservative estimates since we have adopted the view that only the incident cases of diseases related to the risk factors for the 2008 population will be reduced. Our assessment was restricted to new cases of disease avoided, ignoring any reduction in disease amongst those who are already ill. For example, prevalent or existing cases of cardiovascular disease would also benefit from becoming more physically active, eating more fruit and vegetables, losing weight or quitting smoking. Furthermore, we did not measure the likely benefits in future cohorts if our risk factors were further reduced. This was beyond the scope of the project and would require further assumptions to be made, including whether each cohort can be considered independent of another. Nonetheless, it would be anticipated that as prevalence of risk factors reduced through time there would be diminishing marginal returns since there would be fewer people exposed to the risk factors.

Our conservative preference was to use the FCA method as we believe it was more appropriate for our research question. The FCA was shown to halve the relative contribution of workforce productivity gains as a proportion of the total production benefits when compared to using the HCA. We accept that one potential compromise is to argue that workforce production gains will fall somewhere between the HCA and FCA production estimates reported. However, this is probably unnecessary with appropriate uncertainty modelling. We found that the upper level of the UI for the FCA was similar to the point estimate for the HCA regarding workforce production (where the range of uncertainty was much wider).

The most appropriate economic methods for quantifying and valuing household production and leisure time remain an area of continued debate [37]. Potential limitations include the fact that individuals may also perform overlapping activities because of time constraints, such as watching television while minding a child or cooking while listening to a radio. Floro and Miles have reported estimates of overlapping time spent for men and women for the labour market, household work and leisure activities [38]. In brief, about one third of every activity involves at least one other simultaneous activity [38]. Therefore, it can be difficult to estimate changes in the quantity of household production and leisure time precisely.

Finally, we recommend caution in the interpretation of the presented 'opportunity cost savings' because these benefits will only be achieved by the adoption of effective interventions that will certainly have implementation costs attached to them. We did not include intervention costs for this analysis and we have assumed that effective interventions exist to achieve the target reductions in the prevalence of the risk factors. Furthermore, the opportunity cost savings are not estimates of immediately realisable financial savings, but rather estimates of resources used in current practice that could be available for other purposes.

## Conclusions

This study was designed to contribute important new knowledge about the potential impact of a reduction in risk factor related disease on health sector expenditure, workforce production, household production and leisure time. The selection of targets for risk factor prevalence reduction is an important policy decision and a useful approach for future analyses. Modelling Arcadian ideals, together with relying on guidelines/expert opinion, ensured modelled estimates were relevant, and realistic, in the Australian context to future prevention strategies. Importantly, future research providing insights into productivity impacts for individuals with and without risk factors over time would form the basis of more accurate estimates of avoidable production losses associated with ill health, which is currently limited by reliance on cross-sectional data. This work provides useful information to decision makers on estimating the potential benefits of reducing behavioural risk factors in Australia. Similar approaches could be applied in other countries if the data are available.

## Abbreviations

AUD: Australian dollars; ABS: Australian Bureau of Statistics; BMI: body mass index; BoD: burden of disease; CURF: Confidentialised Unit Record Files; DALY: disability adjusted life year; DCIS: Disease Costs and Impact study; FCA: Friction cost approach; GDP: gross domestic product; HCA: Human capital approach; NHS: national health survey; NPV: net present value; PAFs: population attributable risk fractions; UI: uncertainty interval; USD: United States dollars.

## Competing interests

The authors declare that they have no competing interests.

## Authors' contributions

Each author has made substantial contributions to this study and this manuscript. DC and AM designed the study, developed economic models, performed data analyses and supervised project staff. LS contributed to the literature reviews and drafting of the manuscript; TC contributed to the literature reviews, advisory committee meetings and drafting of the manuscript; DP analysed the national health survey data, contributed to advisory committee meetings and drafting of the manuscript; RC contributed to the design of the study, advisory committee meetings, supervision of project staff and drafting of the manuscript. All authors read and approved the final manuscript.

## Authors' information

DC has a clinical background in nursing and holds a Master of Public Health in health economics and health program evaluation. Her PhD was an economic evaluation of stroke prevention interventions. She is currently supported by a joint National Health and Medical Research Council and National Heart Foundation Public Health Research Fellowship (ID 610313) and is the Head of the Public Health division at the National Stroke Research Institute. She has an honorary staff fellowship appointment with Deakin Health Economics (Deakin University) and the Department of Medicine at The University of Melbourne.

AM has a background in health economics, epidemiology and public health and is a fulltime Senior Research Fellow with Deakin Health Economics, Deakin University.

LS is a Research Fellow with Deakin Health Economics, Deakin University and has a Master of Public Health.

TC is a Post-Doctoral Research Fellow at the National Stroke Research Institute and has a background in psychology, research into physical inactivity, cognition and depression.

DP is a Post-Doctoral Research Fellow for the Melbourne School of Population Health at The University of Melbourne and has a background in biology, information technology and epidemiology and biostatistics.

RC is a Professor of Health Economics at Deakin University and has an extensive research and policy background in priority setting and decision-making in health.

## Pre-publication history

The pre-publication history for this paper can be accessed here:

http://www.biomedcentral.com/1471-2458/11/483/prepub

## Supplementary Material

Additional file 1 **Table S1 Summary of unit prices used to estimate household production and leisure time costs**. Table S1 contains the input values (average, low and high) of child care, domestic services and average weekly earnings used to estimate household production and leisure time costs. Table S2 Summary of input parameters and uncertainty ranges for the economic models. Table S2 contains detail of the sources, values, uncertainty distributions and detailed comments relevant to all data inputs used in the modelling. Click here for file
