# Supplementary material for: The societal benefits of reducing six behavioural risk factors: an economic modelling study from Australia
Source: BMC Public Health. 2011 Jun 21;11:483. doi: 10.1186/1471-2458-11-483 (PMC3146859; doi:10.1186/1471-2458-11-483)
Supplement: Additional file 1 — Table S1 Summary of unit prices used to estimate household production and leisure time costs. Table S1 contains the input values (average, low and high) of child care, domestic services and average weekly earnings used to estimate household production and leisure time costs. Table S2 Summary of input parameters and uncertainty ranges for the economic models. Table S2 contains detail of the sources, values, uncertainty distributions and detailed comments relevant to all data inputs used in the modelling. [file 1471-2458-11-483-S1.DOC]

**Additional file**

**Supplemental Tables**

**The societal benefits of reducing six behavioural risk factors: an economic modelling study from Australia**

Dominique A Cadilhac^1,2,3*^, Anne Magnus^1^, Lauren Sheppard^1^,Toby B Cumming^2^, Dora C Pearce^3^, Rob Carter ^1^

^1^Deakin Health Economics, Deakin University, Burwood 3125, Victoria, Australia

^2^National Stroke Research Institute, Heidelberg Heights 3081, Victoria, Australia

^3^The University of Melbourne 3010, Parkville, Victoria, Australia

*Corresponding author

Email addresses:

DAC: [cadilhac@unimelb.edu.au](mailto:cadilhac@unimelb.edu.au)

AM: [anne.magnus@deakin.edu.au](mailto:anne.magnus@deakin.edu.au)

LS: [lauren.sheppard@deakin.edu.au](mailto:lauren.sheppard@deakin.edu.au)

TBC: [tcumming@unimelb.edu.au](mailto:tcumming@unimelb.edu.au)

DCP: [dpearce@unimelb.edu.au](mailto:dpearce@unimelb.edu.au)

RC: [rob.carter@deakin.edu.au](mailto:rob.carter@deakin.edu.au)

Table S1 Summary of unit prices used to estimate household production and leisure time costs

| **Unit Prices** | |  | **Uncertainty Ranges** | | |
| --- | --- | --- | --- | --- | --- |
|  | | **Hourly rate**  **AUD** | **Low** | **High** | |
| **Household production*** | |  |  |  | |
| ***Child care*** | |  |  |  | |
| Unqualified | | $15.24 | $14.65 | $15.79 | |
| Qualified (certificate III level) | | $17.38 | $16.78 | $19.95 | |
|  | |  |  |  | |
| ***Domestic Services*** | |  |  |  | |
| General services (gardening/ housekeeping, laundry) | | $16.13 | $15.72 | $17.29 | |
| Food services (cook) | | $16.40 | $15.72 | $16.53 | |
| Technical, clinical, personal care (nurse attendant/ personal care worker) | | $16.40 | $15.72 | $16.99 | |
| **Leisure time** | | | | | |
| ***Average weekly earnings*** [1] | | | | | |
| % applied [2] | 33% | | 25% | | 50% |
| Males | $10.44 | | $7.84 | | $15.68 |
| Females | $8.79 | | $6.60 | | $13.20 |

*Sources of unit prices: Pay Scale Summaries- Victorian (2005) Award AP840807-FED and Health and Allied Health Private Sector Vic Consolidated Award 1998 [AP783872-FED] (note these pay scale summaries provide current 2008 prices).

**Table S2 Summary of input parameters and uncertainty ranges for the economic models**

| **Data Item** | **Source** | **Values** | **Distribution** | **Comments*** |
| --- | --- | --- | --- | --- |
| ***Workforce Production Gains model*** | | | | |
| Disease or risk factor specific work force participation rate | National Health Survey 2004/05 [3] | Mean , n | Binomial | By 10 age groups and sex. |
| Comparator specific work force participation rate | National Health Survey 2004/05 [3] | Mean , n | Binomial | By 10 age groups and sex. |
| Disease or risk factor specific absenteeism rate | National Health Survey 2004/05 [3] | Mean , SE | Normal | By 10 age groups and sex. Number of days away from work in the last 2 working weeks. |
| Comparator specific absenteeism rate | National Health Survey 2004/05 [3] | Mean , SE | Normal | By 10 age groups and sex. Number of days away from work in the last 2 working weeks. |
| Days worked in a year at full-time status | Assumed | 240 | N/A | 5 days for 48 weeks |
| Employment status full time and part time of diseased or risk factor exposed population | National Health Survey 2004/05 [3] | Mean SE | Normal | By 10 age groups and sex. |
| Days worked in a year at part-time status | Weekly earnings in main job - by hours paid for in main job [1] | 47% work 60 days  34% work 147 days  19% work 192 days | Cumulative | Assuming an 8 hour day and 48 weeks/year worked. |
| Wage multiplier | Pauly [4]  Nicholson [5] Koopmanschap [6] | 0.275 to 1.3 | Uniform | Applied to reflect compensation mechanisms in workplaces. |
| Hiring and Training costs | Dept Treasury and Finance Human Resources Contractor | 15% to 20% of gross wage for persons < 35 years & 35% to 50% for persons > 35 years. | Uniform | Higher rates apply to higher wages |
| Australian average weekly earnings | [1] | Mean , SE | Normal | By 10 age groups and sex. |
| Real Wages Growth | Dept Treasury and Finance internal document | 0.016 | N/A | Introduced to yield consistent comparisons with other DTF projects |
| Friction Period (months) | [6, 7] | 3, 6 | N/A | Varied in sensitivity analysis. |
| Discount rates |  | 0, 0.03, 0.05 | N/A | Varied in sensitivity analysis. |
| Retirement age |  | 65 | N/A | assumed |
| ***Household and Leisure time model*** | | | | |
| Hourly unit prices household and leisure activities | Refer Table 1 | Refer Table 3 | Triangular | Refer Table 3 |
| Hours engaged in household production | Time use Survey 2006 [8] | Mean ,+ - SE | Triangular | Surveyed by gender, age and employment status |
| Hours engaged in leisure activities | Time use Survey 2006 [8] | Mean , +- SE | Triangular | Surveyed by gender, age and employment status |
| Diseased or risk factor specific absenteeism rate | National Health Survey 2004/05 [3] | Mean , SE | Normal | Number of days away from work in the last 2 working weeks. |
| Comparator specific absenteeism rate | National Health Survey 2004/05 [3] | Mean , SE | Normal | Number of days away from work in the last 2 working weeks. |
| Diseased or risk factor specific reported count of days of reduced activity due to ill health | National Health Survey 2004/05  [3] | Mean , SE | Normal | Measured over a 14 day period for persons not in the labour force and over 65 separately. |
| Years of remaining life expectancy for risk factor populations | ABS Life Tables 2004-2006. [9] | M 27.6:41.4  F 35.0:45.5 | Uniform | By gender |

ABS: Australian Bureau of Statistics; N/A: not applicable; SE: Standard error; M: male; F: female

* further details can be found in the online technical research report

[http://www.vichealth.vic.gov.au/~/media/ResourceCentre/PublicationsandResources/Knowledge/Research%20Report_FINAL_July09.ashx](http://www.vichealth.vic.gov.au/~/media/ResourceCentre/PublicationsandResources/Knowledge/Research Report_FINAL_July09.ashx)

**References**

1. Australian Bureau of Statistics: **6302.0 - Average Weekly Earnings, Australia**, May 2008. Canberra: ABS; 2008:36.

2. Shaw WD, Feather P: **Possibilities for including the opportunity cost of time in recreation demand models**. *Land Economics* 1999, 75:592-602.

3. Australian Bureau of Statistics: **4363.0.55.001 - National Health Survey:** Users' Guide - Electronic Publication, 2004-05. Canberra: ABS; 2006:248.

4. Pauly MV, Nicholson S, Xu J, Polsky D, Danzon PM, Murray JF, Berger ML: **A general model of the impact of absenteeism on employers and employees**. *Health Econ* 2002, 11(3):221-231.

5. Nicholson S, Pauly MV, Polsky D, Sharda C, Szrek H, Berger ML: **Measuring the effects of work loss on productivity with team production**. *Health Econ* 2006, 15(2):111-123.

6. Koopmanschap MA, Rutten FF: **A practical guide for calculating indirect costs of disease**. *Pharmacoeconomics* 1996, 10(5):460-466.

7. Koopmanschap MA, Rutten FF, van Ineveld BM, van Roijen L: **The friction cost method for measuring indirect costs of disease**. *J Health Econ* 1995, 14(2):171-189.

8. Australian Bureau of Statistics: **4153.0 - How Australians Use Their Time, 2006**. Canberra: ABS; 2008.

9. Australian Bureau of Statistics: **3302.0.55.001 Life Tables, Australia 2004–2006**. Canberra: ABS; 2007.
